# Regulatory divergence and functional diversification of a c-di-GMP-controlled sigma factor in Actinomycetota

**DOI:** 10.1128/msystems.00352-26

**Published:** 2026-08-07

**Authors:** Joee D. Denis, Govind Chandra, Justine J. Choi, Yves V. Brun, Kelley A. Gallagher

**Affiliations:** 1 Department of Microbiology, Cornell University 5922, Ithaca, New York, USA; 2 Department of Molecular Microbiology, John Innes Centre 15549, Norwich, England, United Kingdom; 3 Département de Microbiologie, Infectiologie et Immunologie, Université de Montréal, Montreal, Canada; Third Institute of Oceanography, Xiamen, China

**Keywords:** Actinomycetota, c-di-GMP signaling, second messenger, sigma, anti-sigma, WhiG, RsiG, development, sporulation, flagella, motility, protein evolution

## Abstract

Members of the σ^28^ family of alternative σ factors typically regulate genes involved in flagellar biosynthesis. However, the only member of the σ^28^ family present in the actinobacterial genus *Streptomyces*, WhiG, controls the differentiation of aerial hyphae into spores. WhiG activity is regulated by the second messenger c-di-GMP, which arms its cognate anti-σ, RsiG, to bind and sequester the σ factor. Understanding WhiG evolution can thus shed light on the diversity of processes regulated by c-di-GMP across the phylum Actinomycetota. Members of Actinomycetota comprise highly diverse filamentous and unicellular, flagellated and non-flagellated species, and the actinobacterial ancestor is predicted to have been motile. Here, our systematic analysis reveals that WhiG homologs are broadly distributed throughout Actinomycetota and form two distinct clades: WhiG1, whose members have retained the ancestral association with the flagellar biosynthesis cluster and are not regulated by an RsiG anti-σ, and WhiG2, whose members are typically regulated by RsiG via c-di-GMP. These findings highlight a phylogenetic split in the regulation of this key transcription factor throughout the phylum. Bioinformatic analysis of WhiG target regulons suggests that this σ factor has significantly diversified in function to control various processes, including motility, chemotaxis, type IV pili synthesis, sporulation, antibiotic biosynthesis, and c-di-GMP metabolism across diverse actinobacterial species.

**IMPORTANCE:**

Mounting global responses to dynamic environmental conditions is a crucial function that bacterial cells must perform. Global regulatory networks are most often studied in individual species, however, understanding how regulatory networks have evolved in distinct bacterial lineages remains an outstanding question. The alternative sigma factor WhiG, which is found in the actinobacterial genus *Streptomyces*, is a dedicated sporulation sigma, yet has long been known to have a close evolutionary relationship to sigma factors that are responsible for regulation of flagellar biosynthesis. Analysis of the distribution of WhiG in the phylum Actinomycetota reveals that homologs typically co-occur with either a flagellar cluster or the c-di-GMP-binding anti-sigma RsiG. Functional predictions of WhiG target genes further reveal that these transcription factors have been co-opted during the evolution of the Actinomycetota to control diverse biological processes.

## INTRODUCTION

The nucleotide second messenger 3′,5′-cyclic diguanylic acid (c-di-GMP) plays a critical role in coordinating global cellular responses to environmental changes (1). Cellular c-di-GMP levels are tightly regulated by the action of diguanylate cyclases (DGCs), which synthesize c-di-GMP, and phosphodiesterases (PDEs), which degrade it (2–4). When present, c-di-GMP can interact with downstream effector proteins, which often modulate target systems involved in fundamental microbial behaviors such as motility, virulence, and biofilm formation (1). In the filamentous bacterial genus *Streptomyces*, which resides in the phylum Actinomycetota*,* c-di-GMP governs complex morphological development, including the formation of vegetative mycelium followed by aerial mycelium production and sporulation (5–7).

**Fig 1:**
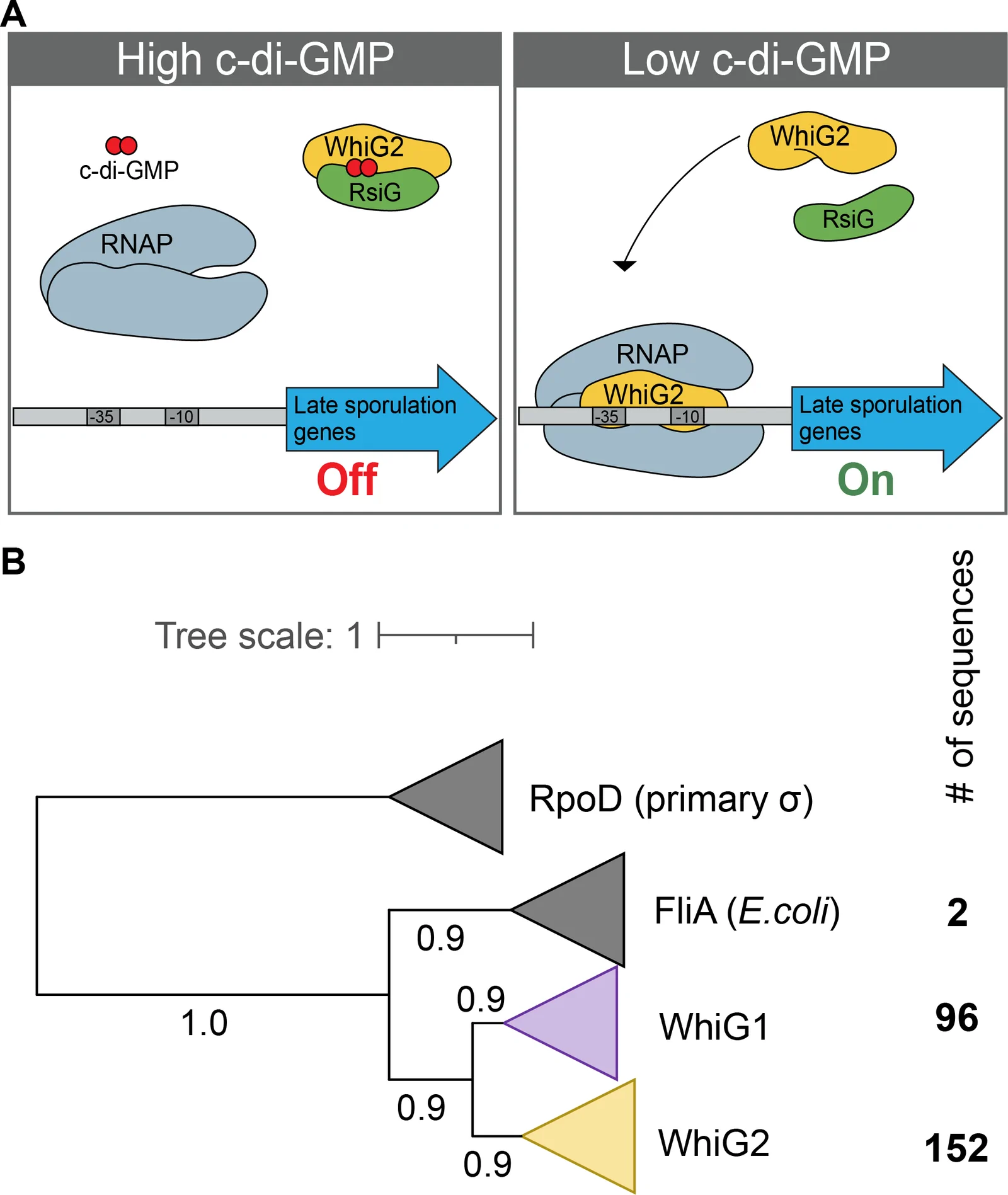
WhiG homologs cluster into two distinct and well-supported subclades. (**A**) Schematic representation of c-di-GMP-dependent regulation of the RsiG-WhiG2 complex in *S. venezuelae*. (Left) At elevated intracellular c-di-GMP levels, the anti-σ RsiG binds WhiG2 via a c-di-GMP dimer, sequestering WhiG2 and preventing its association with RNA polymerase. (Right) At lower intracellular levels of c-di-GMP, the RsiG-(c-di-GMP)_2_-WhiG2 complex disassociates, freeing WhiG2 to associate with RNA polymerase and direct transcription of late sporulation genes. (**B**) Maximum likelihood phylogeny of WhiG homologs found in 673 reference Actinomycetota genomes. Bootstrap values are indicated at the respective nodes based on 100 replicates. The number of WhiG homologs found in each subclade is indicated to the right of each clade. Tree scale is substitutions per site.

We previously showed that c-di-GMP regulates *Streptomyces* development by controlling activity of the σ factor WhiG (8). WhiG is a sporulation-specific σ factor that regulates the expression of three known target genes in the model species *Streptomyces venezuelae*: the developmental regulators *whiH* and *whiI* and a poorly conserved membrane protein of unknown function (*vnz_15005*) (8). *whiH* and *whiI* are also confirmed WhiG targets in the species *Streptomyces coelicolor* (9, 10). WhiH is a GntR-family transcriptional regulator (9), and WhiI is an orphan response regulator (11). Both WhiH and WhiI are expressed during the late stages of *Streptomyces* development (8, 11, 12) and are essential for the proper maturation of aerial hyphae into spores. The precise timing of WhiG activity is thus crucial for proper development. The activity of WhiG is controlled post-translationally by a cognate anti-σ factor, RsiG. To interact with WhiG, RsiG must bind a dimer of c-di-GMP. When bound to RsiG, WhiG is sequestered, leaving its set of target genes off. Cellular levels of c-di-GMP in *S. venezuelae* are high during vegetative growth and drop prior to sporulation (13). Therefore, the relatively high cellular levels of c-di-GMP during vegetative growth favor RsiG-(c-di-GMP)-WhiG complex formation (Fig. 1A). Later in the life cycle, c-di-GMP levels drop, the complex disassociates, and WhiG can then direct RNA polymerase (RNAP) to transcribe its set of target genes (8, 14).

We previously showed that RsiG distribution is limited to the phylum Actinomycetota, where it is most conserved in the families Streptomycetaceae, Geodermatophilaceae, and Pseudonocardiaceae (15). Characteristic c-di-GMP-binding signatures were found in a majority of RsiG sequences, suggesting that binding the second messenger is likely to be a conserved feature of these proteins. In support of this, we showed that the RsiG homolog from *Rubrobacter radiotolerans* forms a complex with WhiG and confirmed these homologs bind two molecules of c-di-GMP in a similar manner to *S. venezuelae* RsiG (15). These findings indicate that RsiG binds c-di-GMP and functions as a cognate anti-σ factor for WhiG in distant actinobacterial species. Phylogenetically, WhiG belongs to the σ^28^ family of alternative factors that regulate flagellum biosynthesis in diverse bacteria, including FliA in members of the phylum Pseudomonadota and SigD in members of the phylum Bacillota (16–18). Flagellar biosynthesis is subject to a regulatory hierarchy that ensures correct assembly of the complex appendage. During early stages of flagellar assembly, FliA/SigD is held inactive by a cognate anti-σ factor, FlgM (19–22). FlgM is not known to bind c-di-GMP and association with its cognate σ is regulated via a distinct mechanism. Specifically, following hook-basal body assembly, FlgM is secreted (23–25), thereby releasing FliA/SigD. Once active, FliA/SigD is responsible for activating the expression of genes required for the late stages of flagellar biosynthesis (26, 27).

Within the phylum Actinomycetota, the function of WhiG homologs has been analyzed in two genera in addition to *Streptomyces*: *Rubrobacter* and *Actinoplanes*. In our previous analysis of *Rubrobacter*, we showed that the WhiG homolog in this genus targets type IV pilus biosynthesis, as well as several PDEs/DGCs, indicating the presence of regulatory feedback loops (15). Interestingly, the actinobacterial species *Actinoplanes missouriensis*, which features a complex developmental life cycle that involves production of sporangia that release motile flagellated zoospores (28, 29), possesses four paralogs of σ factors belonging to the WhiG/FliA family. An RNA-seq analysis by Hashigushi et al. (30) revealed that both developmental and motility-related genes are regulated by these σ factors. The sets of genes de-regulated by the deletion of individual WhiG/FliA genes in *A. missouriensis* were only partially overlapping, indicating non-redundant functions for the paralogs. As a dedicated developmental σ factor, *S. venezuelae* WhiG has functionally diverged from the rest of the clade.

So far, the role of WhiG homologs has been defined in a limited number of actinobacterial taxa and has not been subjected to systematic analysis. Because WhiG is known to be directly controlled by c-di-GMP in the phylogenetically distant actinobacterial genera *Streptomyces* and *Rubrobacter*, understanding the functional evolution of WhiG across the phylum will shed light on the processes controlled by c-di-GMP in these taxa. To examine the evolution of this σ factor, we searched a set of genomes representative of the phylum Actinomycetota to identify WhiG homologs. This analysis revealed that actinobacterial WhiG sequences can be classified into two distinct phylogenetic lineages, which we named WhiG1 and WhiG2. WhiG1 sequences, which include those that are found in the zoospore-producing genus *Actinoplanes,* are co-conserved with the presence of the flagellar biosynthesis cluster, indicating that these homologs may regulate flagellar biosynthesis in diverse actinobacterial species. WhiG1 homologs are not able to form a complex with (c-di-GMP)-RsiG. By contrast, WhiG2 sequences, which include those found in *Streptomyces*, are likely to be regulated by c-di-GMP via RsiG partners. In support of these observations, our bioinformatic analysis predicts that chemotaxis and motility genes are frequently found in WhiG regulons. Along with these genes, we predict that WhiG homologs regulate genes involved in transcriptional regulation, c-di-GMP metabolism, and specialized metabolite biosynthesis. Overall, our analyses indicate a phylogenetically predictable divergence in how actinobacterial WhiG homologs are regulated, as well as significant diversification in the function of WhiG through the evolution of Actinomycetota.

## RESULTS

### Actinobacterial WhiG homologs form two distinct sub-clades

Our previous work assessing the distribution of the c-di-GMP-binding anti-σ factor RsiG revealed that it is found in diverse actinobacterial taxa and always co-occurs with a putative WhiG homolog (15). However, this previous study did not address the distribution of WhiG homologs, and it has remained unclear if most actinobacterial WhiG homologs are likely to be regulated by RsiG partners. In addition, the identification of four WhiG paralogs in *A. missouriensis* (30) suggests that these proteins have been subject to expansions during evolution of the phylum though the overall extent of this is unknown. Hence, to assess the distribution of WhiG within the Actinomycetota, we searched 673 reference/representative Actinomycetota genomes (Table S1) available at GenBank using the *S. venezuelae* WhiG (WhiG*_Sv_*, Vnz_26215) sequence as a query in a BLAST search. Identified sequences were then aligned with a set of reference sequences representing major σ^70^ families (31) (Table S2), and a phylogenetic tree was constructed. A well-supported clade that included all reference sequences from the FliA/SigD family was used to define the set of WhiG homologs (248 total). Of the 673 genomes analyzed, approximately 30% (187 genomes) possess a WhiG homolog. To refine the evolutionary relationships among the identified sequences, an actinobacterial WhiG phylogeny was then constructed (Fig. 1B). The initial bifurcation in the phylogeny reveals two distinct and well-supported sub-clades, which were designated as the WhiG1 clade (96 sequences) and the WhiG2 clade (152 sequences, including WhiG*_Sv_*).

### RsiG forms a complex with WhiG2, but not WhiG1 homologs

**Fig 2:**
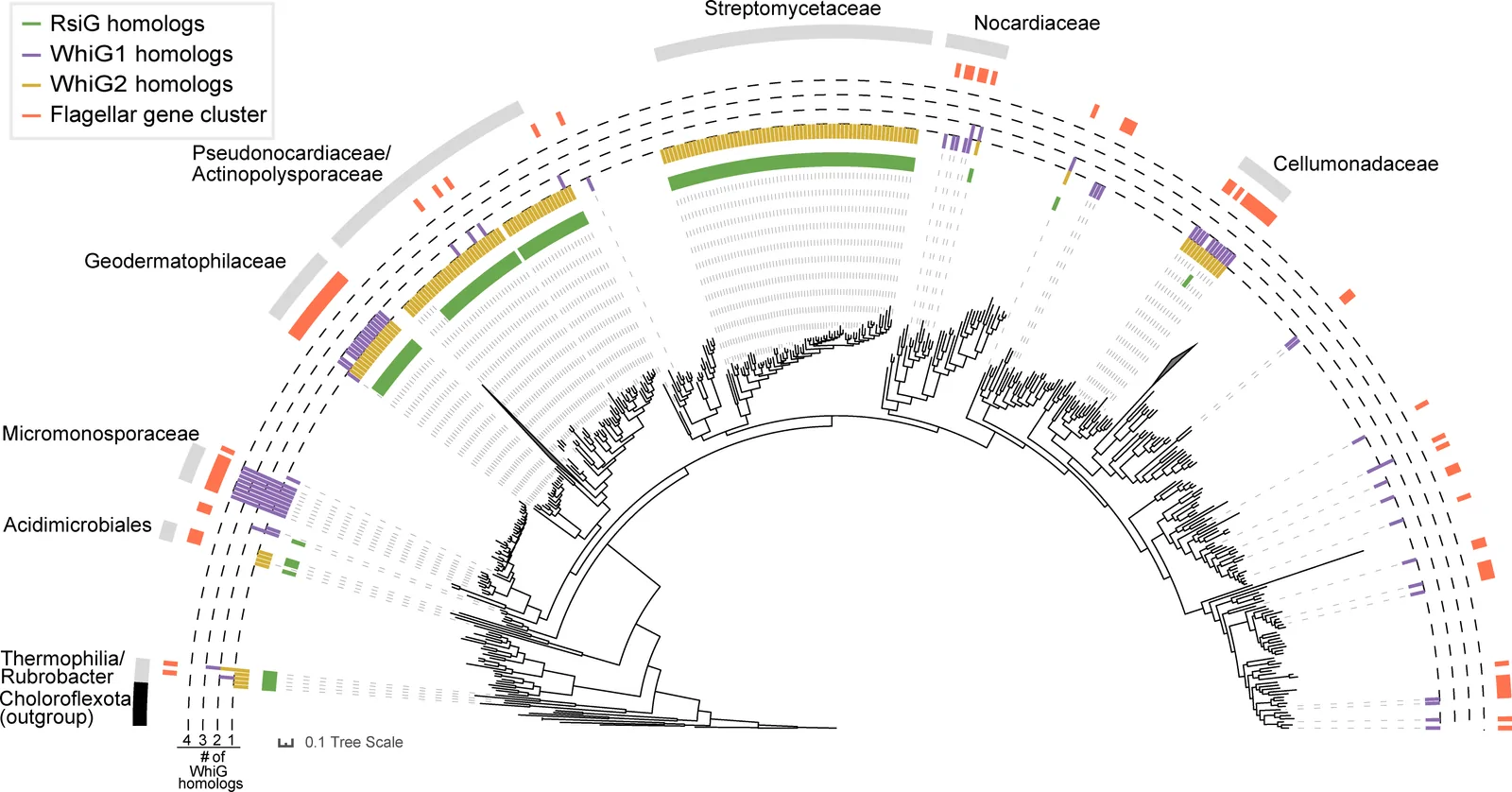
Distribution of WhiG1, WhiG2, and RsiG homologs and flagellar clusters across phylum Actinomycetota. A maximum likelihood tree of 673 representative Actinomycetota genomes is shown based on 37 concatenated housekeeping genes that were identified and aligned using PhyloSift (32). Sequences derived from ten Chloroflexota genomes were used as an outgroup (indicated in black). Genomes possessing an RsiG homolog are indicated by green bars. The presence of WhiG homologs is indicated by purple bars (WhiG1) and yellow bars (WhiG2); bar height reflects the number of WhiG homologs within each genome. Genomes possessing a flagellar gene cluster are indicated with orange bars. Taxonomic families with at least 50% of members containing a WhiG homolog are indicated by the gray arcs. Clades comprised of species from order Corynebacteriales (194 genomes) and family Bifidobacteriaceae (37 genomes) are represented as gray triangles, as no members of these taxa possess WhiG, RsiG, or flagellar gene homologs. Tree scale is substitutions per site.

To assess the co-occurrence of the WhiG homologs with RsiG, we mapped the identified sequences onto a housekeeping phylogeny that reflects the evolutionary history of the 673 actinobacterial genomes that were included in the search (15) (Fig. 2). The presence of a WhiG2 homolog significantly correlated with the presence of a RsiG homolog (Mantel’s correlation coefficient *r* = 0.88, *P* = 0.001), but not with WhiG1 (Mantel’s correlation coefficient *r* = 0.09, *P* = 0.002) sequences. This co-distribution pattern suggested that WhiG2 homologs may be regulated by RsiG and c-di-GMP, as seen in the *S. venezuelae* model, whereas WhiG1 homologs may not form a complex with these molecules.

**Fig 3:**
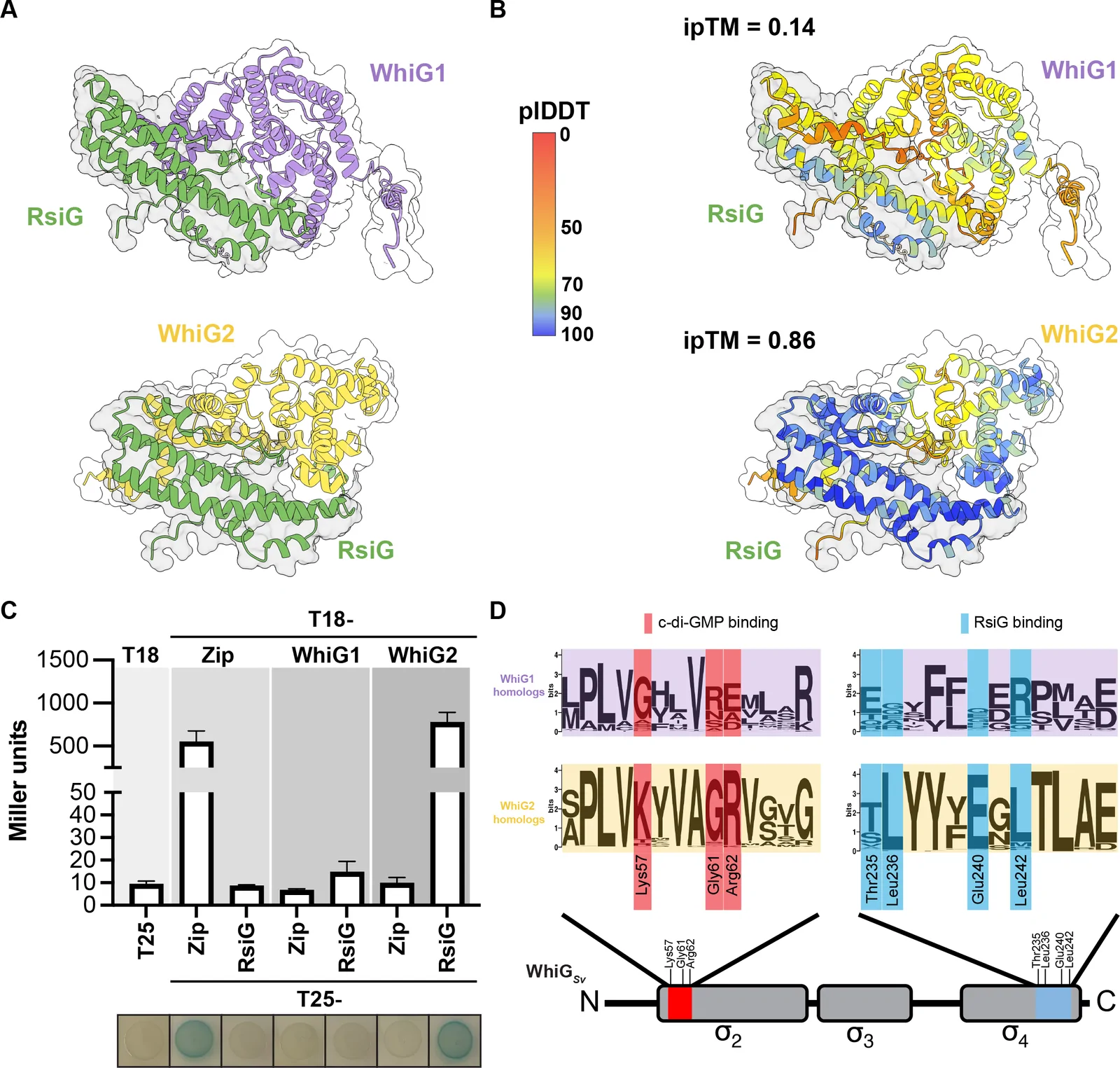
RsiG homologs interact only with WhiG2 homologs. (**A**) Alphafold3 models of RsiG*_Go_* (green) in complex with (top) WhiG1*_Go_* (purple) and (bottom) WhiG2*_Go_* (yellow). (**B**) Alphafold3 models of the interaction of RsiG*_Go_* with (top) WhiG1*_Go_* and (bottom) WhiG2_Go_ colored based on the structural confidence measure predicted lDDT (local distance difference test). Confidence in each complex is indicated by interface predicted template (ipTM) score. (**C**) Bacterial two-hybrid analysis to investigate the interaction between RsiG*_Go_* with WhiG1*_Go_* and WhiG2*_Go_*. The listed pairs of constructs were transformed into the BACTH reporter strain *E. coli* BTH101. Strains expressing fusions of both adenylate cyclase domains to the leucine zipper domain of GCN4 (Zip) served as a positive control, whereas strains expressing fusions of one adenylate cyclase domain to a Zip domain and the other to RsiG*_Go_*, WhiG1*_Go_,* or WhiG2*_Go_* served as negative controls. Results are the average of three replicate cultures derived from the same single colony. Error bars represent SEM. (**D**) Schematic depiction of WhiG domain organization below sequence logos depicting amino acid sequence conservation in the two clades of WhiG homologs at key sites within the σ_2_ and σ_4_ domains. Residues critical to the RsiG*_Sv_*-(c-di-GMP)_2_-WhiG*_Sv_* complex are noted below sequence logos. Residues that are critical for the interaction of WhiG*_Sv_* with c-di-GMP are highlighted in red and residues involved in the interaction of WhiG*_Sv_* with RsiG are highlighted in blue. Two separate multiple sequence alignments of WhiG1 homologs and WhiG2 homologs were used to generate the sequence logos using Weblogo (36).

To further explore the relationship between RsiG and the two clades of WhiG homologs, we employed Alphafold3 (AF3) modeling (33). AF3 predicts a high-confidence interaction (ipTM = 0.80) between the *S. venezuelae* RsiG (RsiG*_Sv_*, Vnz_19430) and WhiG*_Sv_* proteins (where high confidence is defined as an ipTM score ≥0.80), even without the inclusion of c-di-GMP in the model. We therefore sought to predict these interactions in a representative genome from the family *Geodermatophilaceae*, where RsiG, WhiG1, and WhiG2 co-occur with high frequency (Fig. 2). Members of this taxonomic family are frequently isolated from soil environments, have been described to grow as rods or rudimentary hyphae, and produce flagellated motile spores (34). An AF3 model of the RsiG and WhiG2 homologs encoded in the genome of *Geodermatophilus obscurus* DSM 43160 (RsiG*_Go_* and WhiG2*_Go_*, Gobs_4624 and Gobs_3994, respectively)*,* the type species of the genus (35), predicts a high confidence interaction (ipTM = 0.86), while the model of RsiG*_Go_* with the WhiG1 clade homolog from the same species (WhiG1*_Go_*, Gobs_0987) does not predict complex formation (ipTM = 0.14) (Fig. 3A and B). To validate these predictions *in vivo*, we assayed these proteins for interaction using a bacterial adenylate cyclase two-hybrid (BACTH) system. This confirmed that RsiG*_Go_* and WhiG2*_Go_* interact, in striking contrast to RsiG*_Go_* and WhiG1*_Go_*, which do not (Fig. 3C).

To examine if our findings using the *G. obscurus* proteins are likely to extend to most members of the WhiG1 and WhiG2 clades, we assessed the conservation of residues critical for the interaction between the σ factor and the c-di-GMP ligand, as well as between the σ factor and anti-σ factor, by generating separate multiple sequence alignments for each clade. The structure of the *S. venezuelae* RsiG-(c-di-GMP)-WhiG complex revealed that three residues from the WhiG*_Sv_* σ_2_ domain make direct contacts with the c-di-GMP dimer: Lys57, Gly61, and Arg62 (8). Conservation of Gly61, which packs against one of the ribose groups of c-di-GMP, is likely particularly important for the interaction, as any other side chain would cause a steric clash (8). Sequence logos made using these alignments illustrate that, indeed, all three residues are well conserved among WhiG2, but not WhiG1 homologs (Fig. 3D). Similarly, residues in the σ_4_ domain of WhiG*_Sv_* (including Thr235, Leu236, Glu240, and Leu242) that make direct contacts with RsiG*_Sv_* (8) are well conserved in the alignment of the WhiG2 clade but are highly variable in the WhiG1 clade (Fig. 3D). Taken together, results of the co-distribution analysis, AF3-predicted structures, BACTH assay, and conservation of residues required for complex formation strongly suggest that RsiG, when present, is likely to physically interact with and regulate the activity of WhiG2 clade members via c-di-GMP but is unlikely to regulate WhiG1 clade homologs.

### WhiG1 sequences are co-distributed with the flagellar biosynthesis cluster

Notably, we almost always observe at least one WhiG1 homolog encoded in representative/reference genomes of the genera *Actinoplanes* and *Geodermatophilus* (Fig. 2), both of which are known to have members that produce flagellated zoospores (28, 34). Based on the phylogenetic relationship of WhiG with flagellar σ factors (i.e., *Salmonella* FliA and *Bacillus* SigD) (16) and recent findings that WhiG homologs in *A. missouriensis* control flagellar biosynthesis (30), we hypothesized that WhiG1 homologs may be associated with this cluster. To examine the co-occurrence of flagellar genes and WhiG1 in Actinomycetota, we conducted a cblaster (37) search of the set of actinobacterial representative genomes using the *A. missouriensis* 431 (NBRC 102363) cluster (38) as query (Table S3) and mapping the presence of the cluster onto the housekeeping phylogeny (Fig. 2). We detected the presence of the flagellar cluster in 12% of genomes, with the highest conservation of the cluster observed in the genera *Actinoplanes* (Micromonosporaceae)*, Geodermatophilus* (Geodermatophilaceae), and *Cellulomonas* (Cellulomonadaceae). This distribution of the flagellar gene cluster is largely in agreement with a previous analysis of the evolution of flagella in Actinomycetota (39). Strikingly, in 81% of cases where we observed a flagellar cluster, a WhiG1 clade homolog was also present (Mantel’s correlation coefficient *r* = 0.84, *P* = 0.001). This was not the case for WhiG2 clade homologs, which are present in only 46% of genomes with a flagellar cluster (Mantel’s correlation coefficient *r* = 0.14, *P* = 0.001). Furthermore, 62% of WhiG1 clade homologs are encoded within or near to the flagellar cluster itself (defined as being encoded within five genes of a gene within the cluster). These observations suggest that WhiG1 clade homologs may be functionally similar to the FliA/SigD family members found outside of the phylum Actinomycetota.

### WhiG1 and WhiG2 homologs recognize similar promoters

**Fig 4:**
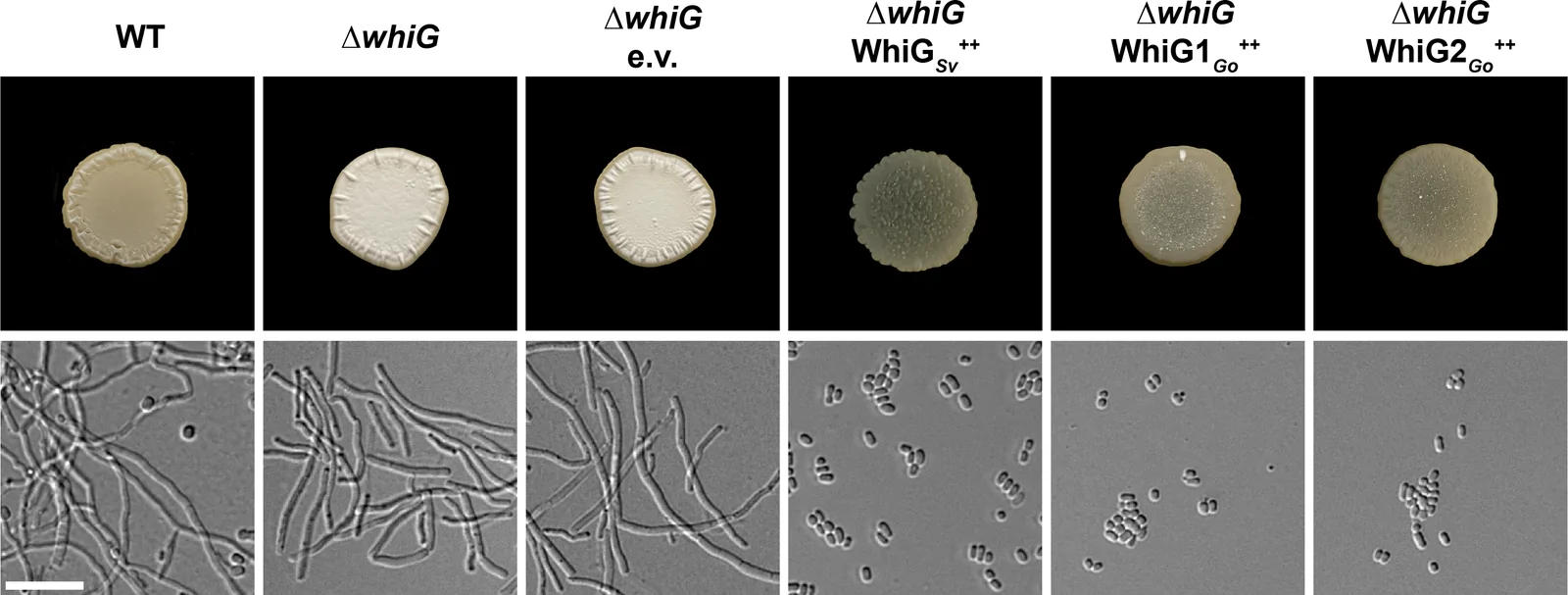
Overexpression of *G. obscurus whiG1* or *whiG2* homologs in *S. venezuelae* recapitulates the native *whiG* overexpression phenotype. (Top) Macrocolonies of wild type (WT) *S. venezuelae*, the *whiG* mutant, the *whiG* mutant carrying the corresponding empty vector (e.v.), the *whiG* mutant overexpressing *whiG_Sv_*, the *whiG* mutant overexpressing *whiG1_Go_*, and the *whiG* mutant overexpressing *whiG2_Go_*, photographed after 48 h of growth on Difco Nutrient Agar (DNA). (Bottom) Representative DIC micrographs of the same strains imaged after 48 h of growth on DNA. Scale bar: 10 μm.

We next wondered if WhiG1 and WhiG2 homologs are likely to recognize distinct or similar promoters. Target sequences for WhiG homologs have been the subject of previous studies in three individual actinobacterial genomes. In the first of these, the WhiG target sequence in *S. venezuelae* was identified using ChIP-seq (8). Second, the previous analysis of *R. radiotolerans* used bioinformatic prediction combined with 5′ triphosphate end capture transcription start site mapping to predict 12 WhiG target sites (15). Finally, the WhiG target sequence in *A. missouriensis* was recently predicted by RNA-seq (30). Based on the phylogeny of WhiG homologs established here, we now know that the *S. venezuelae* and *R. radiotolerans* homologs belong to the WhiG2 clade, while the *A. missouriensis* homologs belong to the WhiG1 clade. Despite this, the reported target sequences for all are highly similar to the well-established consensus (40) for the FliA/SigD family. Therefore, to test if WhiG1 and WhiG2 are able to recognize distinct promoter sequences, we relied on the striking hypersporulation phenotype in *S. venezuelae* that results from overexpression of *whiG_Sv_* in a *ΔwhiG* background (41). Hypersporulation in this species results in the differentiation of vegetative hyphae into spores, which leads to a bald-like colony phenotype. Hypersporulation is also apparent in micrographs, where most of the biomass is visible as spores (8). We placed the *whiG1_Go_* and *whiG2_Go_* under the control of the constitutive ermE* promoter in *S. venezuelae ΔwhiG*. The introduction of either homolog from *G. obscurus* resulted in a hypersporulation phenotype similar to the effect of overexpressing the native *whiG* sequence (Fig. 4). The ability to recapitulate the *whiG_Sv_* overexpression phenotype suggests that both homologs are able to recognize similar promoter sequences. Furthermore, we analyzed conservation of key residues that make base-specific contacts with the promoter region based on a structure of the *Pseudomonas aeruginosa* FliA-RNAP open promoter complex (42). Key residues for interaction with DNA were highly conserved among both clades of WhiG homologs (Fig S1).

### Bioinformatic prediction of actinobacterial WhiG homolog regulons

To gain further insights into the functional diversification of these σ factors, we performed a bioinformatic search to identify likely WhiG target promoters. We searched for matches to the conserved WhiG1/2 consensus (−35 XT(C/A)AX; −10 GCCGA) in the intergenic regions of genomes with a WhiG homolog. Positive hits were required to lie within 200 bp upstream of a start codon, and one mismatch was allowed in the first two bases of the −10, which have been observed to be more variable (8, 15, 30). We limited this search to taxa within Actinomycetota with high conservation of WhiG homologs (defined as genera with at least two representatives in which a WhiG homolog is found in >80% of genomes).

**Fig 5:**
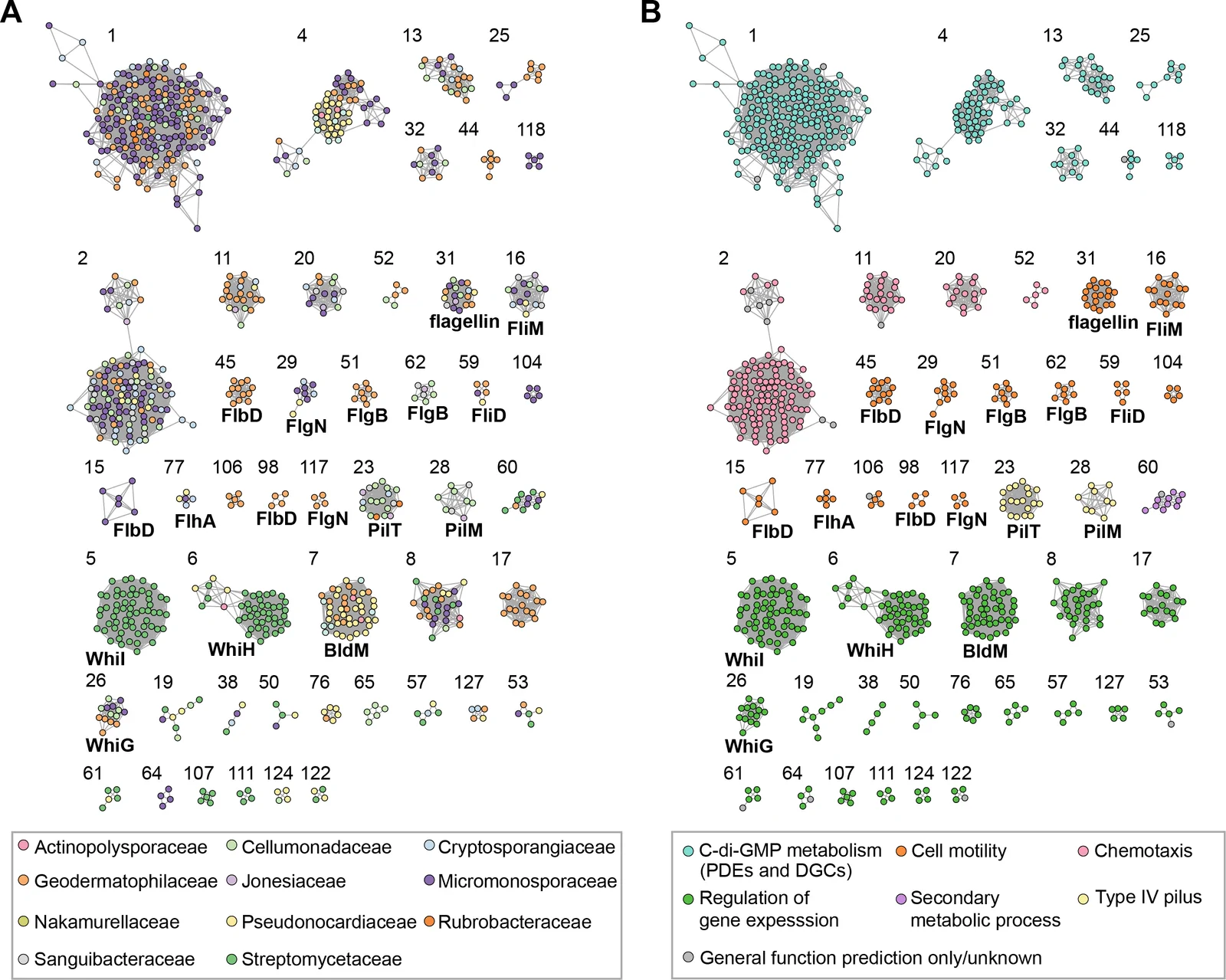
Subset of sequence similarity network of the predicted phylum-wide WhiG regulon. WhiG promoters (as defined in Materials and Methods) were identified in genera with at least two representatives in our dataset and in which WhiG homologs were present in >80% of genomes. Genes located immediately downstream of these promoters were subjected to all-versus-all BLAST and clustered using EFI-EST (43). The resulting network was filtered to include categories of biological interest (discussed in text) and visualized in Cytoscape version 3.10.3 (44). A version of this network including all predicted target genes can be found in Fig S2. Clusters are numbered according to yFiles organic layout algorithm (45) and given a gene annotation if >80% of nodes share the same annotation. Nodes represent individual genes, and edges denote BLAST pairwise similarity scores <1E−35. Nodes are colored according to taxonomic family (**A**) or associated biological process based on GO terms (**B**).

To analyze the identified targets, we constructed a sequence similarity network (41, 43), where protein sequences sharing ≥35% identity are connected via edges (Fig. 5; Fig S2; Table S4). Conserved components of WhiG regulons are therefore grouped into clusters in the network. The resulting sequence similarity network is comprised of 131 total clusters. Notably, one of these, cluster 26, is comprised entirely of WhiG homologs, indicating that in some species WhiG regulates its own promoter. Strikingly, the known *S. venezuelae* WhiG targets WhiI (Vnz_28820) and WhiH (Vnz_27205) were found in two of the largest clusters (clusters 5 and 6). Both of these clusters are primarily comprised of hits from Streptomycetaceae genomes (Fig. 5; Fig S2), suggesting that these are highly conserved as WhiG targets within the family. Thirteen additional clusters are exclusively found in *Streptomyces* genomes, which may represent previously unknown and/or species-specific targets. Clusters comprised of genes found only in genomes from a single taxonomic family were also observed for Geodermatophilaceae, Micromonosporaceae, Pseudonocardiaceae, and Cellumonadaceae. In contrast, several clusters, including some of the largest clusters, are comprised of targets derived from genomes representing considerable taxonomic diversity. For example, the largest cluster in the network (cluster 1) has representatives from eight distinct actinobacterial families (Fig. 5).

Functional predictions of each of the putative targets were performed using Gene Ontology (GO) assignments (Table S5), which revealed significant diversification of WhiG regulons. In addition, we generated HMM profiles from the sequences within each cluster. These HMM profiles were then searched against the *S. venezuelae* genome (Table S6) to identify homologs in a well-characterized reference system. Of clusters with a predicted biological process, most were involved in regulation of gene expression. Interestingly, using the HMM profile constructed using sequences from cluster 7, the top hit in *S. venezuelae* was BldM (Vnz_22005). BldM is an orphan atypical response regulator that in *Streptomyces* forms a heterodimer with WhiI, and together the two proteins control the expression of approximately 22 genes (11). BldM is predicted to be a WhiG target in genomes representing disparate actinobacterial families, but not in any *Streptomyces* genomes. Outside of *Streptomyces*, it is unknown if BldM and WhiI form heterodimers. Additional targets that are predicted to be involved in regulation of gene expression include clusters where a majority of members are annotated as response regulators (cluster 5, which includes WhiI, cluster 6, which includes WhiH, and clusters 8, 17, 57, 64, 124), σ factors (cluster 26, which includes WhiG, and cluster 127), WhiB-like (cluster 107), LacI-family (clusters 19, 50), helix-turn-helix motif-containing (clusters 65, 61), as well as transcriptional regulators belonging to the TetR (cluster 111), AfsR (cluster 122), IclR (cluster 76), and ROK (cluster 53) families.

**Fig 6:**
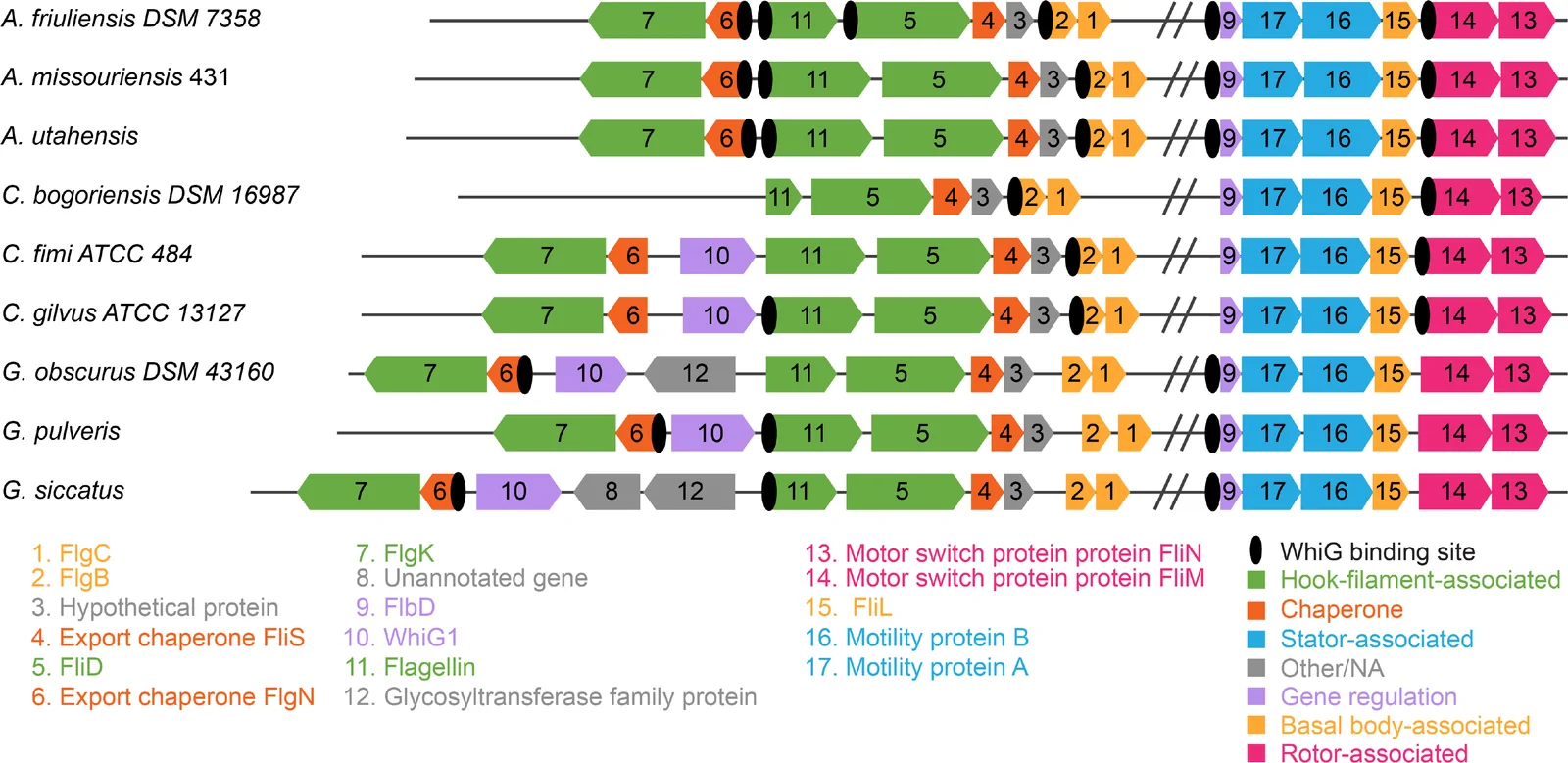
Predicted WhiG target promoters in flagellar gene cluster of *Actinoplanes friuliensis* DSM 7358, *Actinoplanes missouriensis* 431, *Actinoplanes utahensis*, *Cellulomonas bogoriensis* DSM 16987, *Cellulomonas fimi* ATCC 484, *Cellulomonas gilvus* ATCC 13127, *Geodermatophilus obscurus* DSM 43160, *Geodermatophilus pulveris*, and *Geodermatophilus siccatus*. Genes in the flagellar gene cluster are shown as schematics with the predicted gene products listed below with the same color coding. Positions of the predicted WhiG target promoters are shown as black ovals.

In addition to the regulation of gene expression, one of the most conserved functional predictions we observed were the enzymes responsible for c-di-GMP metabolism. Seven clusters within our network (clusters 1, 4, 13, 25, 32, 44, 118) comprised of 307 putative target genes are predicted to function as DGC/PDE enzymes (Fig. 5). These putative targets are found in eight distinct actinobacterial families, making c-di-GMP metabolism the most widespread function of WhiG targets across the phylum. Despite this, we only observed two WhiG-binding sites in front of genes encoding PDEs/DGCs in the family Streptomycetaceae, two EAL-domain containing proteins in the species *Streptomyces autolyticus* and *Streptomyces melanosporofaciens* (Fig. 5; Table S4). In addition, for most genomes included in the analysis that possess genes for flagellar biosynthesis, we observed putative WhiG-binding sites embedded within these gene clusters. Frequently, this occurs multiple times within a flagellar gene cluster, indicating that WhiG homologs potentially regulate the transcription of multiple operons in these clusters (Fig. 6). Additional notable biological process categories identified include genes predicted to be involved in chemotaxis (clusters 2, 11, 20, 52) in members of seven distinct actinobacterial families (Fig. 5; Table S4), and type IV pilus biosynthesis pathways (clusters 23, 28) in members of the Cellumonadaceae, Cryptosporangiaceae, Sanguibacteraceae, Rubrobacteraceae, and Jonesiaceae (Fig. 5). Genes involved in specialized metabolite biosynthesis (cluster 60) are also predicted targets in several families, including the Micromonosporaceae, Pseudonocardiaceae, Geodermatophilaceae, and Streptomycetaceae. These targets include genes that are predicted to have core functions in specialized metabolite biosynthesis, including non-ribosomal peptide synthetases and polyketide synthases (Table S4).

## DISCUSSION

Despite being broadly distributed in bacteria, to date the only member of the flagellar-associated family of σ factors known to be directly controlled via c-di-GMP are those found in the phylum Actinomycetota (15), exemplified by WhiG from the genus *Streptomyces*. The work presented here reveals that actinobacterial WhiG homologs can be classified into at least two distinct groups based on phylogenetic inference. WhiG1 homologs are unable to associate with RsiG partners and have retained the ancestral association with the flagellar biosynthesis cluster. WhiG2 homologs, which include all of those found in the genus *Streptomyces*, are likely largely regulated by RsiG partners via c-di-GMP. The bifurcation of the actinobacterial WhiG phylogeny into two sister clades suggests that an ancient duplication event occurred during early evolution of the Actinomycetota which was followed by divergence in the regulatory mechanism controlling σ activity. The identity of a putative anti-σ partner for WhiG1 homologs remains unclear, especially given the notable absence of FlgM, the cognate anti-σ of canonical flagellar σ factors FliA/SigD, in almost all flagellar clusters found in Actinomycetota (39). The actinobacterial ancestor is predicted to have been motile, and the flagellar cluster was likely largely inherited vertically throughout the phylum Actinomycetota though loss events occurred along many lineages (39). Therefore, FlgM was likely lost as a part of the flagellar cluster during early evolution of the phylum, and its absence may indicate an alternative, as-yet unidentified mechanism for regulation of the late stages of flagellar biosynthesis in these species.

So far, the function of these homologs has been explored in a limited number of species that are relatively scattered throughout the phylum Actinomycetota. Previous work in *A. missouriensis*, which possesses four homologs that all belong to the WhiG1 clade, has shown that their regulons are only partially overlapping (30), suggesting that even WhiG homologs within the same clade can exhibit a degree of functional divergence. Although the overexpression of both *whiG1_Go_* and *whiG2_Go_* was able to recapitulate the effect of overexpressing the native *whiG* sequence in *S. venezuelae*, it is unclear if these are responsible for the regulation of distinct or overlapping regulons when they co-occur in the same organism. Regardless of the extent of regulon overlap, when WhiG1 and WhiG2 homologs co-occur they are likely to be regulated by distinct input signals, mediated by c-di-GMP_2_-RsiG (for WhiG2) and by an unknown mechanism (for WhiG1).

We observed a spectrum of taxon-specificity for predicted WhiG target genes, with some targets found in genomes spanning the actinobacterial phylogeny, and other targets restricted to individual taxonomic families. One of the most abundant functional categories we identified were target genes predicted to encode DGCs/PDEs, the enzymes responsible for synthesis and degradation of c-di-GMP itself. These target genes were found in genomes that are very broadly phylogenetically distributed, representing all but one of the taxonomic families that were included in the search. Many of these predicted targets are from genomes where only a (c-di-GMP)-RsiG-regulated WhiG2 homolog is found, suggesting the presence of regulatory feedback loops responsible for control of c-di-GMP metabolism. Interestingly, another transcription factor that is directly regulated by c-di-GMP, called BldD, is known to repress transcription of multiple DGCs in *Streptomyces* species (14, 46–48). BldD is a repressor that must form a complex with c-di-GMP to bind its target genes (46, 49). In *S. venezuelae,* BldD is active during vegetative growth, while WhiG2 becomes active during the maturation of aerial hyphae into spores. This suggests that for *Streptomyces,* there may be more direct regulation of c-di-GMP via the expression of genes encoding DGCs during earlier stages of the life cycle.

Genes involved in motility and chemotaxis were frequently identified as predicted WhiG targets. This includes most species in our search that possess a flagellar gene cluster. The conservation of components of chemosensory pathways and structural proteins of the flagellar filament in these regulons suggests that the role of actinobacterial WhiG homologs is likely to be required for later stages of flagellar biosynthesis similar to the role of SigD/FliA in the Bacillota and Pseudomonadota (26, 27). Our findings are in alignment with the previous study in *A. missouriensis*, in which WhiG1 homologs (referred to there as “FliA”) are shown to target genes involved in motility (30). From studies of these phylogenetically distant phyla, c-di-GMP itself is known to directly regulate the process of motility at multiple layers including the regulation of gene expression, flagellar assembly, motor rotation, and chemotaxis (50). Our analysis also revealed the presence of WhiG binding sites within clusters responsible for the biosynthesis of type IV pili. Type IV pili are surface appendages that are involved in diverse functions including competence, twitching motility, surface sensing, and microcolony formation (51, 52). Notably, in *A. missouriensis*, type IV pili are present on zoospores in addition to flagella and are required for adhesion to hydrophobic solid surfaces (53). Predicted binding sites within these clusters were previously confirmed in *A. missouriensis* (30) and *R. radiotolerans* (15). Our analysis suggests that the regulation of type IV pilus biosynthesis by WhiG is conserved across *Rubrobacter*. Like motility, type IV pilus biosynthesis in gram-negative bacteria is known to be a c-di-GMP-regulated process, and c-di-GMP can affect multiple levels of this regulation (54, 55). Thus, the direct and multi-layered regulation of both motility and type IV pilus biosynthesis via c-di-GMP appears to be broadly conserved across diverse bacterial phyla.

One of the most frequent functional categories of genes that we predict to be a part of WhiG regulons are transcription factors. In several cases, this includes WhiG itself (cluster 26), which is consistent with observations in *Salmonella* (56) and *Bacillus* (57) that FliA/SigD control the transcription of *fliA/sigD*. Additionally, our analysis revealed that WhiI is a target of WhiG2 homologs in almost all (97%) of the *Streptomyces* genomes included in our search. WhiH homologs were also found as predicted targets in a majority (67%) of *Streptomyces* genomes. In *S. venezuelae*, WhiH is known to control a large regulon of late sporulation genes (58). Similarly, *S. venezuelae* WhiI in complex with BldM controls a regulon of ~40 sporulation genes (11). Thus, by activating transcription of *whiH* and *whiI, Streptomyces* WhiG homologs are likely to indirectly control large sets of genes involved in late stages of sporulation. Interestingly, BldM homologs also appear in our SSN (cluster 7) although none of these hits were found in *Streptomyces* genomes. The separation of BldM and WhiI expression has a clear function in *Streptomyces*: prior to WhiI expression, BldM forms a homodimer and regulates genes that are required for earlier stages of the life cycle. Once *whiI* expression is activated by WhiG, BldM forms a heterodimer and controls a second set of genes required for spore maturation (11). Outside of *Streptomyces*, it is unknown if BldM and WhiI are able to form a functional heterodimer. The appearance of BldM as a major target outside of *Streptomyces* provides an example of rewiring of related transcriptional networks through the evolution of Actinomycetota. Additional uncharacterized transcription factors that we have identified during our analysis may represent novel downstream regulators in the WhiG-regulated pathways, such as development and motility, and will be the subject of future study.

Given the high degree of conservation of the signature c-di-GMP-binding motif(s) among RsiG homologs (15), it is likely that most of these homologs are able to bind the second messenger. Thus, the identification of WhiG2 regulons are, by extension, predictions of the processes that are directly regulated by c-di-GMP signaling. Interestingly, there does appear to be some functional overlap between WhiG1 and WhiG2, as we frequently found predicted WhiG binding sites in front of PDEs and DGCs in genomes that possess single homologs belonging to either clade. Overall, this work points to significant diversification of WhiG regulation and function over the course of actinobacterial evolution. Further work is needed to understand whether WhiG1 and WhiG2 regulons overlap when the two proteins co-exist.

## MATERIALS AND METHODS

### Actinomycetota phylogeny

A search set of 673 Actinomycetota genomes was chosen based on annotation as “reference” or “representative” at GenBank and use in a previous analysis of RsiG distribution (15), along with 10 genomes from the phylum Chloroflexota that serve as an outgroup (Table S1). The Chloroflexota genome *Anaerolinea thermophila* UNI-1 was used to root the tree. Amino acid sequences of 37 conserved housekeeping genes were automatically identified, aligned, and concatenated using Phylosift (32). Model selection was performed using SMS (59) implemented at http://www.atgc-montpellier.fr/phyml/ (60) which resulted in selection of an LG substitution model with γ-distributed rate variation between sites. Phylogenetic reconstruction was performed by RAxML version 8.2.10 (61) with 100 rapid bootstrap replicates to assess node support. The tree was visualized and formatted using iTOL (62). Taxonomic assignments were based on the taxonomy database maintained by the NCBI (https://www.ncbi.nlm.nih.gov/Taxonomy/Browser/wwwtax.cgi).

### WhiG homolog identification and alignment

WhiG homologs were identified by a BLAST search (*e*-value cutoff = 0.001) of the same set of 673 annotated Actinomycetota genomes using the WhiG*_Sv_* sequence as a query. This permissive search resulted in a set of 3,836 candidate sequences. 1,769 of these sequences that are very unlikely to belong to the σ^28^ subfamily were removed from analysis on the basis of returning a protein other than WhiG*_Sv_* when used in a BLAST search of the *S. venezuelae* genome. The remaining hits included σ sequences known to fall outside of the σ^28^ subfamily. To further refine the set of hits, candidate sequences were aligned using MAFFT (63) with a set of 16 reference sequences that includes representatives from all σ^70^-family groups (31) (Table S2). The resulting multiple sequence alignment was then used to construct an approximately-maximum-likelihood phylogeny using FastTree 2 (64). A well-supported clade was identified that included all reference sequences from the σ^28^ family and no reference sequences from other σ^70^-family subgroups (i.e., groups 1, 2, and 4). This clade was therefore used to define the set of WhiG homologs (248 total). To refine the phylogeny for the set of confirmed actinobacterial WhiG homologs, these sequences were re-aligned using CLUSTAL Omega (65). Model selection was performed using ModelTest-NG (66) which resulted in the selection of an LG substitution model with γ-distributed rate variation between sites. Phylogenetic reconstruction was performed by RAxML version 8.2.10 (61) with 100 rapid bootstrap replicates to assess node support. The tree was visualized and formatted using iTOL (62). To visualize conservation of residues involved in interaction with c-di-GMP or RsiG, WhiG1 (*n* = 96) or WhiG2 (*n* = 152) homologs were extracted from the alignment and sequence logos generated using Weblogo (36).

### Flagellar cluster identification

To identify genomes that possessed the genetic potential to produce a flagellum, a search for the flagellar gene cluster in our local database of 673 reference Actinomycetota genomes was performed using cblaster (37) with default parameters (minimum query coverage = 50%, minimum sequence identity = 30%, and a maximum distance between any two hits = 20 kbp). Thirty-three flagellar genes (Table S3) from *Actinoplanes missouriensis* 431 (38) were used in the query. Genomes were considered to possess a flagellar gene cluster if they contained at least 30% of the query genes (*e*-value cutoff = 0.01).

### Statistical analysis

Partial Mantel tests with the Pearson method were used to determine correlations between the presence of WhiG1 or WhiG2 homologs and RsiG homologs or the flagellar cluster while controlling for the influence of phylogenetic distance. Mantel tests were performed in R version 4.5.1 (67) using the package vegan (68) and set to 999 permutations. A phylogenetic distance matrix was obtained from the maximum likelihood tree of 673 representative actinobacterial species using the “cophenetic.phylo” function of the package ape (69).

### Protein modeling

Protein complex predictions of WhiG1*_Go_* and WhiG2*_Go_* with RsiG were generated using AlphaFold3 (33). Confidence in the complexes was measured by interface predicted template modeling (ipTM) score (33). ChimeraX version 1.8 (70) was used to generate structural representations.

### Bacterial strains, plasmids, and media

Strains, plasmids, and oligonucleotides used in this study are listed in Table S7. *E. coli* strain DH5α was used for plasmid propagation and grown on LB or LB agar at 37°C. The *dam dsm hsdS E. coli* strain ET12576 containing pUZ8002 (71) was used to conjugate plasmids into *S. venezuelae* (72, 73). *S. venezuelae* strains were grown on Difco Nutrient Agar (DNA) or in Difco Nutrient Broth (DNB). To study development on solid media, strains were grown overnight in DNB and 10 μL spotted onto DNA plates and incubated for 48 h. Where required for selection, the following antibiotics were added to growth media: 100 μg/mL carbenicillin, 50 μg/mL kanamycin, and/or 25 μg/mL hygromycin.

### Bacterial adenylate cyclase two-hybrid assays

*E. coli* codon-optimized versions of the *rsiG*, *whiG1*, and *whiG2* genes from *G. obscurus* DSM 43160 were generated by gene synthesis (Twist Biosciences). These were PCR-amplified with primers JD07-JD18 (Table S7) and assembled into pUT18 and pKT25 (8, 15, 74) digested with XbaI and KpnI to create plasmids pJD001-pJD012 (Table S7). *E. coli* BTH101 was then co-transformed with the “T18” and “T25” fusion plasmids. pUT18 and pKT25 empty constructs were used as a negative control. β-Galactosidase activity was assayed in biological triplicate.

### Overexpression of proteins in *S. venezuelae*

*Streptomyces* codon-optimized versions of the *whiG1* and *whiG2* genes from *G. obscurus* DSM 43160 were generated by gene synthesis (Twist Biosciences). These were assembled into NdeI/HindIII-cut pIJ10257 (75) to generate plasmids pJC001 and pJC002. This construction results in *whiG1* or *whiG2* being placed under the control of the strong constitutive ermE* promoter. Overexpression plasmids were introduced into the *S. venezuelae* Δ*whiG* mutant by conjugation.

### Light microscopy

Strains were grown on DNA plates until fully differentiated (48 h), and a coverslip pressing was created by spotting 10 μL of 20% glycerol onto the colony and transferring to a 1% agarose pad. Strains were visualized using a Nikon Eclipse Ti2 inverted microscope fitted with a CFI60 Plan Apochromat Lambda D Oil 60× objective. Still images were collected using NIS-Elements (Nikon) and analyzed using the Fiji Software package (https://fiji.sc/).

### Prediction of WhiG regulons

In genomes possessing a WhiG homolog, we searched intergenic regions within 200 bp upstream of a start codon for the conserved WhiG1/2 promoter consensus (−35 XT(C/A)AX; −10 GCCGA) sequences. The spacer that separates the −35 and −10 was set at a length of 16 bases for our search query. The −10 region was required to contain CGA at positions 3–5 and up to one mismatch was allowed in positions 1–2. This search resulted in 7,199 matches. The amino acid sequences of the downstream genes of the matched motifs were uploaded to EFI-EST (43) to generate a sequence similarity network (SSN) (edge *e*-value cutoff = 1.0E−5). The generated SSN was further filtered to retain clusters comprised of >3 nodes, resulting in 131 clusters encompassing 1,490 nodes (Fig S2; Table S4). Sequence similarity networks were visualized using Cytoscape (44). Clusters were numbered according to yFiles organic layout algorithm (45).

Functional annotation of WhiG targets was performed by Blast2GO (76), which employs the Gene Ontology Consortium (77, 78) database to assign GO terms based on existing annotation associations. GO terms were categorized into broad functional categories using ancestor charts and parental terms (Table S5). Nodes with broad or generic annotations (e.g., GO: 0016787 hydrolase activity) were analyzed with InterProScan (79) to identify functional domains and then sorted into a functional annotation category (e.g., EAL domain sorted to “c-di-GMP metabolism”). Nodes still lacking a specific annotation or with no annotation available were classified as “General function prediction only/unknown.” In addition, predicted WhiG target sequences were binned by cluster number and aligned using MUSCLE (80). Resulting alignments were used to create hidden Markov model (HMM) profiles with “hmmerbuild” from the HMMER suite (hmmer.org, version 3.4). These profiles were queried against the *Streptomyces venezuelae* genome (GCA_001886595.1) using “hmmsearch” of the HMMER suite.

To illustrate the conservation of WhiG targets in the flagellar gene cluster, nine representative species from three genera with high levels of WhiG1 conservation were selected. FlaGs (Flanking Genes) (81) was used to identify sets of homologous flanking genes and create a graphical visualization of the flagellar gene cluster. Genomes were aligned according to the two most frequently targeted flagellar genes, flagellin (*n* = 18) and FlbD (*n* = 18).

## Data Availability

A list of reference genomes with accessions, WhiG/RsiG/flagellar-cluster hit lists, regulon prediction outputs, and HMM search results are provided in the supplemental material. SSN files, newick files, alignment files used to create phylogenetic trees and HMM profiles, and AF3 model files are available at https://doi.org/10.6084/m9.figshare.33006122. A docker image for carrying out regulon predictions is available at https://hub.docker.com/r/streptomyces/gbkscan2024, and the Perl script which carries out the searches is available at https://github.com/jdd232/gbkscan2024.

## SUPPLEMENTAL MATERIAL

Supplemental material msystems.00352-26-s0001.docx Supplemental figures and tables.

Table S1 msystems.00352-26-s0002.csv Reference Actinomycetota and Chloroflexota genomes.

Table S4 msystems.00352-26-s0003.csv Predicted WhiG targets in Actinomycetota.

Table S6 msystems.00352-26-s0004.csv Results of HMM profile search of *Streptomyces venezuelae* genome using WhiG regulon clusters.

ASM does not own the copyrights to Supplemental Material that may be linked to, or accessed through, an article. The authors have granted ASM a non-exclusive, world-wide license to publish the Supplemental Material files. Please contact the corresponding author directly for reuse.
