# Supplementary material for: Regulatory divergence and functional diversification of a c-di-GMP-controlled sigma factor in Actinomycetota
Source: mSystems. 2026 Aug 7;11(9):e00352-26. doi: 10.1128/msystems.00352-26 (PMC13595943; doi:10.1128/msystems.00352-26)
Supplement: Supplementary file 1 [file msystems.00352-26-s0001.docx]

**Fig S1** Residues in WhiG1 and WhiG2 homologs involved in interaction with DNA are generally well-conserved. (Left) Schematic depiction of WhiG domain organization with regions numbered as previously defined (2). (Right) Sequence logos depicting amino acid sequence conservation among all WhiG homologs (i.e. includes members of WhiG1 and WhiG2 clades) from genomes of family Geodermatophilaceae. The non-template (NT) and template (T) strands of the promoter DNA encompassing the -35 and -10 regions are depicted. Asterisks highlight residues within the σ_2,_ σ_3,_ and σ_4_ domains known from the structure of the *P. aeruginosa* FliA-RNAP open promoter complex (3) to make sequence-specific interactions between the σ and bases in the promoter with the same colored asterisks.

**Fig S2** Full sequence similarity network of the predicted phylum-wide WhiG regulon. WhiG promoters were identified in genera with at least two representatives in our dataset and in which WhiG homologs were present in >80% of genomes. Genes located immediately downstream of these promoters were subjected to all-versus-all BLAST and clustered using EFI-EST (4). The resulting network is visualized in Cytoscape version 3. 10.3 (5). Clusters are numbered according to yFiles organic layout algorithm (6). Nodes represent individual genes, and edges denote BLAST pairwise similarity scores <1E-35. Nodes are colored according to taxonomic family (A) or associated biological process based on GO terms (B).

**Table S1.** Reference Actinomycetota and Chloroflexota genomes used in this study. The number of WhiG1, WhiG2 and/or RsiG homologs found in each genome is indicated. The presence of the flagellar cluster is indicated with a ‘1’, and absence of the flagellar cluster is indicated with a ‘0’.

See separate file

**Table S2.** Reference σ^70^-family sequences used in phylogenetic analysis

| **UniProt Accession** | **Protein** | **σ^70^ subgroup** | **Organism** |
| --- | --- | --- | --- |
| P00579 | RpoD | Group 1 | *Escherichia coli* |
| P52324 | RpoD | Group 1 | *Caulobacter vibrioidescrescentus* |
| P17531 | RpoD | Group 1 | *Myxococcus xanthus* |
| P13445 | RpoS | Group 2 | *Escherichia coli* |
| P45684 | RpoS | Group 2 | *Pseudomonas aeruginosa* |
| P0AEM6 | FliA | Group 3 | *Escherichia coli* |
| P10726 | SigD | Group 3 | *Bacillus subtilis* |
| Q9KQD4 | FliA | Group 3 | *Vibrio cholera* |
| P0AGB3 | RpoH | Group 3 | *Escherichia coli* |
| P06574 | SigB | Group 3 | *Bacillus subtilis* |
| P07860 | SigF | Group 3 | *Bacillus subtilis* |
| P12254 | SigK | Group 3 | *Bacillus subtilis* |
| F2R2N1 | SigR | Group 4 | *Streptomyces venezuelae* |
| H8F0N6 | SigE | Group 4 | *Mycobacterium tuberculosis* |
| Q06909 | CarQ | Group 4 | *Myxococcus xanthus* |
| Q06198 | AlgU | Group 4 | *Pseudomonas aeruginosa* |

**Table S3.** Flagellar genes in *Actinoplanes missouriensis* 431 used as queries for cblaster search in Actinomycetota genomes

| **Gene** | **Gene Product** |
| --- | --- |
| AMIS76150 | FliW |
| AMIS76160 | FlgL |
| AMIS76170 | FlgK |
| AMIS76180 | FlgN |
| AMIS76190 | FliC |
| AMIS76200 | FliD |
| AMIS76210 | FliS |
| AMIS76220 | Unknown |
| AMIS76230 | FlgB |
| AMIS76240 | FlgC |
| AMIS76250 | FliE |
| AMIS76260 | FliF |
| AMIS76270 | FliG |
| AMIS76280 | FliH |
| AMIS76290 | FliI |
| AMIS76300 | Unknown |
| AMIS76310 | LytA |
| AMIS76320 | FliK |
| AMIS76330 | Unknown |
| AMIS76340 | Unknown |
| AMIS76350 | FlhA |
| AMIS76360 | FlhB |
| AMIS76370 | FliR |
| AMIS76380 | FliP |
| AMIS76390 | FliO |
| AMIS76400 | FliN |
| AMIS76410 | FliM |
| AMIS76420 | FliL |
| AMIS76430 | MotB |
| AMIS76440 | MotA |
| AMIS76450 | FlbD |
| AMIS76460 | FlgE |
| AMIS76470 | FlgD |

**Table S4.** Predicted WhiG targets in Actinomycetota

See separate file

**Table S5.** GO term assignment sorted into categories of biological associated process

| **GO term** | **Biological associated process** |
| --- | --- |
| GO:0052621 (diguanylate cyclase activity) | **c-di-GMP metabolism** |
| GO:0071111 (cyclic-guanylate-specific phosphodiesterase activity) |  |
| GO:0003924 (GTPase activity) | catalytic activity (**General function prediction only/unknown**) |
| GO:0016491 (oxidoreductase activity) |  |
| GO:0016740 (transferase activity) |  |
| GO:0016787 (hydrolase activity) |  |
| GO:0016853 (isomerase activity) |  |
| GO:0020037 (heme binding) |  |
| GO:0140096 (catalytic activity, acting on a protein) |  |
| GO:0140097 (catalytic activity, acting on DNA) |  |
| GO:0140098 (catalytic activity, acting on RNA) |  |
| GO:0048870 (cell motility) | **Cell motility** |
| GO:0005737 (cytoplasm) | cellular component (**General function prediction only/unknown**) |
| GO:0005886 (plasma membrane) |  |
| GO:0016020 (membrane) |  |
| GO:0044283 (small molecule biosynthetic process) |  |
| GO:0110165 (cellular anatomical structure) |  |
| GO:0009987 (cellular process) |  |
| GO:1901135 (carbohydrate derivative metabolic process) |  |
| GO:0006575 (modified amino acid metabolic process) |  |
| GO:0006935 (chemotaxis) | **Chemotaxis** |
| GO:0003677 (DNA binding) | molecular function (**General function prediction only/unknown**) |
| GO:0005515 (protein binding) |  |
| GO:0035438 (cyclic-di-GMP binding) |  |
| GO:0140657 (ATP-dependent activity) |  |
| NA | NA (**General function prediction only/unknown**) |
| GO:0005975 (carbohydrate metabolic process) | **Primary metabolic process** |
| GO:0006520 (amino acid metabolic process) |  |
| GO:0006629 (lipid metabolic process) |  |
| GO:0030163 (protein catabolic process) |  |
| GO:0036211 (protein modification process) |  |
| GO:0006355 (regulation of DNA-templated transcription) | **Regulation of gene expression** |
| GO:0140110 (transcription regulator activity) |  |
| GO:0044550 (secondary metabolite biosynthetic process) | **Secondary metabolite biosynthesis** |
| GO:0006412 (translation) | **Translation** |
| GO:0045182 (translation regulator activity) |  |
| GO:0006810 (transport) | **Transport** |
| GO:0043683 (type IV pilus assembly) | **Type IV pilus** |

**Table S6.** Results of HMM profile search of *Streptomyces venezuelae* genome using WhiG regulon clusters

See separate file

**Table S7.** Strains, plasmids, and primers used in this study

| **Strains** | **Relevant genotype/notes** | **Reference** |
| --- | --- | --- |
| ***E. coli*** |  |  |
| DH5α | F– φ80*lac*ZΔM15 Δ(*lac*ZYA-*argF*)U169 *rec*A1 *end*A1 *hsd*R17(rK–,mK+) *pho*A *sup*E44 λ– *thi*-1 *gyr*A96 *rel*A1 | Invitrogen |
| BTH101 | F– *cya*-99 *ara*D139 *gal*E15 *gal*K16 *rps*L1 (*Strr*) *hsd*R2 *mcr*A1 *mcr*B1 | Karimova et al., 1998 |
| ET12567(pUZ8002) | ET12567 carrying helper plasmid pUZ8002 | Paget et al., 1999 |
| ***S. venezuelae*** |  |  |
| ATCC10712 | wild type | Gallagher et al., 2020 |
| SV6 | Δ*whiG*::apr | Gallagher et al., 2020 |
| SV6-pIJ10257 | Δ*whiG*::apr with pIJ10257 integrated at the ΦBT1 attachment site | Gallagher et al., 2020 |
| SV6-pIJ10902 | Δ*whiG*::apr with pIJ10902 integrated at the ΦBT1 attachment site | Gallagher et al., 2020 |
| SV6-pJC001 | Δ*whiG*::apr with pJC001 integrated at the ΦBT1 attachment site | This work |
| SV6-pJC002 | Δ*whiG*::apr with pJC002 integrated at the ΦBT1 attachment site | This work |

| **Plasmid** | **Relevant genotype/comments** | **Source** |
| --- | --- | --- |
| pKT25 | Two-hybrid plasmid, N-terminal cyaAT25 fusion (Kan^R^) | Karimova et al., 1998 |
| pKNT25 | Two-hybrid plasmid, C-terminal cyaAT25 fusion (Kan^R^) | Karimova et al., 1998 |
| pUT18 | Two-hybrid plasmid, C-terminal cyaAT25 fusion (Amp^R^) | Karimova et al., 1998 |
| pUTI8C | Two-hybrid plasmid, N-terminal cyaAT25 fusion (Amp^R^) | Karimova et al., 1998 |
| pKT25-zip | A derivative of pKT25 in which the leucine zipper of GCN4 is genetically fused in frame to the T25 fragment | Karimova et al., 1998 |
| pUT18C-zip | A derivative of pUT18C in which the leucine zipper of GCN4 is genetically fused in frame to the T18 fragment | Karimova et al., 1998 |
| pIJ10257 | φBT1 attP-int derived integration vector for the conjugal transfer of DNA from E. coli to *Streptomyces* (HygR) with the ermE* promoter driving constitutive expression | Tschowri et al., 2014 |
| pIJ10902 | pIJ10257 with the ermE* promoter driving *whiG_Sv_* expression | Gallagher et al., 2020 |
| pJD001 | pUT18 carrying *whiG2_Go_* | This work |
| pJD002 | pUT18C carrying *whiG1_Go_* | This work |
| pJD003 | pUT18 carrying *whiG1_Go_* | This work |
| pJD004 | pUT18C carrying *whiG1_Go_* | This work |
| pJD005 | pUT18 carrying *rsiG_Go_* | This work |
| pJD006 | pUT18C carrying *rsiG_Go_* | This work |
| pJD007 | pKT25 carrying *whiG2_Go_* | This work |
| pJD008 | pKNT25 carrying *whiG2_Go_* | This work |
| pJD009 | pKT25 carrying *whiG1_Go_* | This work |
| pJD010 | pKNT25 carrying *whiG1_Go_* | This work |
| pJD011 | pT25 carrying *rsiG_Go_* | This work |
| pJD012 | pNT25 carrying *rsiG_Go_* | This work |
| pJC001 | pIJ10257 with the ermE* promoter driving *whiG1_Go_* expression | This work |
| pJC002 | pIJ10257 with the ermE* promoter driving *whiG2_Go_* expression | This work |

| **Primers** | **5’ sequence** |
| --- | --- |
| JD07 | CTGCAGGTCGACTATGACCAAAGCGGCGAT |
| JD08 | TGAATTCGAGCTCGGAGCGATATCAGCCAGTTTG |
| JD09 | CTGCAGGTCGACTATGCGTTCTAGCGCGAGC |
| JD10 | TGAATTCGAGCTCGGCGCGCTACGACCGGTAGC |
| JD11 | CTGCAGGTCGACTATGACCCCGGAACCGACC |
| JD12 | TGAATTCGAGCTCGGACGACCTTCATCAGCCAG |
| JD13 | GCTGCAGGGTCGGACTACCAAAGCGGCGATC |
| JD14 | TTCTTAGTTACTTAGGAGCGATATCAGCCAGTTTG |
| JD15 | GCTCGAGGGTCGACTATGCGTTCTAGCGCGAGC |
| JD16 | TTCTTAGTTACTTAGGCGCGCTACGACCGGTAGC |
| JD17 | GCTCGAGGGTCGACTATGACCCCGGAACCGACC |
| JD18 | TTCTTAGTTACTTAGGACCTTCATCAGCCAG |

**Bibliography**

1.	Karimova G, Pidoux J, Ullmann A, Ladant D. 1998. A bacterial two-hybrid system based on a reconstituted signal transduction pathway. Proc Natl Acad Sci USA 95:5752–5756.

2.	Paget M. 2015. Bacterial Sigma Factors and Anti-Sigma Factors: Structure, Function and Distribution. Biomolecules 5:1245–1265.

3.	Sheenu, Kumar V, Sahoo PK, Kandiah E, Jain D. 2026. Structural insights into σ28-dependent transcription initiation and its regulation by anti-sigma factor in *Pseudomonas aeruginosa*. Nucleic Acids Research 54:gkaf1414.

4.	Gerlt JA, Bouvier JT, Davidson DB, Imker HJ, Sadkhin B, Slater DR, Whalen KL. 2015. Enzyme Function Initiative-Enzyme Similarity Tool (EFI-EST): A web tool for generating protein sequence similarity networks. Biochimica et Biophysica Acta (BBA) - Proteins and Proteomics 1854:1019–1037.

5.	Shannon P, Markiel A, Ozier O, Baliga NS, Wang JT, Ramage D, Amin N, Schwikowski B, Ideker T. 2003. Cytoscape: A Software Environment for Integrated Models of Biomolecular Interaction Networks. Genome Res 13:2498–2504.

6.	Wiese R, Eiglsperger M, Kaufmann M. 2004. yFiles — Visualization and Automatic Layout of Graphs, p. 173–191. *In* Jünger, M, Mutzel, P (eds.), Graph Drawing Software. Springer Berlin Heidelberg, Berlin, Heidelberg.
